# Collaborative strategies for implementing NCD “best buys” in the eastern Mediterranean and Africa: addressing challenges, creating opportunities, and charting the way forward

**DOI:** 10.3389/fpubh.2025.1653043

**Published:** 2025-09-01

**Authors:** Randa K. Saad, Asmus Hammerich, Adelard Kakunze, Yousef Khader, Haitham Bashier, Magid Al-Gunaid, Mohannad Al Nsour, Eman Abdelaziz Ahmed Rashad Dabou, Imran Rashid Rangraze

**Affiliations:** ^1^Research and Policy, Center of Excellence for Applied Epidemiology, Global Health Development| Eastern Mediterranean Public Health Network, Amman, Jordan; ^2^Department of Non-communicable Diseases and Mental Health, World Health Organization Regional Office for the Eastern Mediterranean, Cairo, Egypt; ^3^Non-communicable Diseases, Injuries and Mental Health Africa Centres for Disease Control and Prevention, Addis Ababa, Ethiopia; ^4^Department of Public Health, Jordan University of Science and Technology, Irbid, Jordan; ^5^Public Health Emergency Management Center, Global Health Development| Eastern Mediterranean Public Health Network, Amman, Jordan; ^6^Department of Public Health, Global Health Development| Eastern Mediterranean Public Health Network, Amman, Jordan; ^7^Executive Office, Global Health Development| Eastern Mediterranean Public Health Network, Amman, Jordan; ^8^RAK Medical and Health Sciences University, Ras Al Khaimah, United Arab Emirates

**Keywords:** Eastern Mediterranean Region, Africa, NCDS, best buys, 5×5 framework

## Abstract

Noncommunicable diseases (NCDs) are an increasing public health challenge in both the Eastern Mediterranean Region (EMR) and Africa, where they contribute to premature mortality and overall disease burden. Despite the availability of cost-effective “Best Buy” interventions, implementation across these regions has been uneven due to various barriers, including limited resources, inadequate multi-sectoral collaboration, and competing health priorities. These challenges are compounded by emerging public health threats like mental health disorders and environmental factors such as air pollution, recently integrated into the expanded “5×5” framework for NCD prevention and management. This paper presents findings from a roundtable held during EMPHNET’s 8th Biennial Regional Conference, which focused on strategies to implement and scale up “Best Buy” interventions in the EMR and Africa. The roundtable gathered regional and global experts to examine successful NCD prevention efforts, explore implementation barriers, and highlight collaboration opportunities. Discussions centered on the need for tailored, context-specific interventions, stronger multi-sectoral partnerships, and increased political commitment to address the growing NCD burden. Sustainable financing was emphasized, with recommendations for establishing innovative funding mechanisms, such as regional NCD and mental health-related funds. Building workforce capacity was highlighted as critical to improving NCD management in resource-constrained settings, particularly through task-shifting models and NCD integration into training programs like the Field Epidemiology Training Program. The discussions underscored the urgency of adopting a whole-of-system approach to addressing NCDs, leveraging lessons from the COVID-19 pandemic to strengthen health systems, especially primary health care, and integrate NCD prevention efforts into broader health agendas across both regions.

## 1 Introduction

Noncommunicable diseases (NCDs) present a significant public health challenge in the Eastern Mediterranean Region (EMR) and Africa, requiring urgent and coordinated responses. The EMR has the highest rate of premature mortality due to one of the four main NCDs, standing at 24.5% (UI: 16.7–34.0), which is higher than that in any other region (1). While this figure reflects a 13% decline since 2000, it lags behind the global reduction of around 22% over the same period (2). In Africa, NCDs are also becoming the leading cause of death. Though communicable diseases have historically dominated public health priorities, NCDs accounted for 37% of all deaths in 2019, up from 24% in 2000 (1). Africa now faces a triple burden of disease, with NCDs, communicable diseases, and injuries contributing to the overall mortality. Of particular concern is the mental health burden, as mental, neurological, and substance use disorders account for over 6% of total disability-adjusted life years, affecting more than 116 million individuals in Africa (3).

The 66th World Health Assembly (WHA66, May 2013) endorsed the Global Action Plan for the Prevention and Control of NCDs (GAP-NCDs) 2013–2020, which has since been extended until 2030 (4, 5). Central to this plan is the implementation of cost-effective interventions known as “Best Buys.” These are evidence-based, cost-effective interventions, typically costing ≤$100 per healthy life year gained (6), that target the four key modifiable NCD risk factors: tobacco use, harmful use of alcohol, unhealthy diets, and physical inactivity. This approach is referred to as the “4×4 framework,” four diseases and four shared risk factors (4). Best Buys are essential tools for Member States to achieve the nine voluntary global targets for NCD prevention and control through the six objectives of the GAP-NCDs (4, 6). Moreover, Best Buys equip health systems to better respond to the needs of individuals living with, or at risk of, major NCDs (6).

In 2023, the Best Buy interventions were updated at the 76th World Health Assembly, expanding the list to 28 interventions. This update retained the “4×4” structure but introduced a “5×5” framework, which adds mental health as a fifth disease area and includes air pollution and other emerging priorities as additional risk factors (7).

The Eastern Mediterranean Public Health Network (EMPHNET) is committed to promoting the implementation of these “Best Buys” and other effective interventions. This commitment is outlined in EMPHNET’s NCDs Operational Guide (8). In alignment with this mandate, this paper presents a thematic synthesis of insights gained during a roundtable held at EMPHNET’s 8th Biennial Regional Conference, titled Collaborative Strategies for Implementing NCD “Best Buys” in the Eastern Mediterranean and Africa: Addressing Challenges, Creating Opportunities, and Charting the Way Forward. The objectives of the roundtable were to highlight successful regional, continental, and global successes that demonstrate the potential of Best Buy interventions, identify common barriers to implementation within the EMR and African contexts, and explore opportunities and collaborative recommendations that would help countries in Africa and the EMR effectively adopt and scale up these interventions to address the growing burden of NCDs. The findings presented in this paper are derived from a structured synthesis of key themes emerging from the roundtable discussion, which brought together experts and stakeholders from both regions.

## 2 Roundtable description

The roundtable was conducted in English and included three expert presentations. Dr. Patricia Richter, Acting Chief of the Global Public Health Systems Branch at the U. S. Centers for Disease Control and Prevention (CDC), discussed global commitments to addressing NCDs. She highlighted the economic burden of NCDs and emphasized the role of modifiable risk factors such as tobacco use, unhealthy diets, and physical inactivity in driving this global health crisis. She also discussed the uneven implementation of “Best Buys” interventions across countries, underscoring the need for a whole-of-system approach to achieve Sustainable Development Goals (SDGs) related to NCDs.

Dr. Asmus Hammerich, Director of Noncommunicable Diseases and Mental Health (NMH) at WHO-EMRO, followed with a presentation introducing the “5×5” framework. He called for stronger national leadership and emphasized the importance of multi-sectoral collaboration in effectively and efficiently addressing NCDs, particularly in resource-limited settings.

The final presentation by Dr. Adelard Kakunze, Unit Lead for NCDs, Injuries, and Mental Health at Africa CDC, provided an overview of Africa CDC’s evolving role, particularly during the COVID-19 pandemic, in addressing NCDs and mental health. He outlined Africa CDC’s six strategic priorities, including strengthening health systems, advocating for political commitment, and improving access to essential medicines and technologies.

The presentations were followed by an interactive Q&A session, moderated by Dr. Randa Saad, Senior Technical Specialist at EMPHNET, where participants discussed challenges, opportunities, and recommendations for advancing NCD prevention and control in both regions. Around 50 individuals participated, including policymakers, public health professionals, Field Epidemiology Training Program (FETP) graduates, residents, researchers, and other health professionals.

## 3 Literature review approach

To support and contextualize the themes discussed during the roundtable, a targeted literature review was conducted. The objective of this review was to identify recent, policy-relevant evidence and guidance documents related to the implementation of WHO-recommended “Best Buy” interventions, the 5×5 NCD framework, and broader strategies for NCD prevention and control in the EMR and Africa.

The review focused on gray literature, technical documents, and peer-reviewed publications. Searches were conducted in PubMed and Google Scholar, as well as official repositories of the WHO, the WHO Regional Office for the Eastern Mediterranean (EMRO, and Africa CDC) The following keywords were used: “noncommunicable diseases,” “NCD Best Buys,” “5×5 framework,” “health system strengthening,” “Africa CDC strategy,” “multi-sectoral collaboration,” “Eastern Mediterranean Region,” and “NCD financing.”

Sources published between 2010 and 2024 were included, with a focus on WHO guidance documents, high-impact academic journals, and regional policy papers. Documents were selected based on their relevance to at least one of the key thematic areas discussed during the roundtable.

## 4 Findings

### 4.1 The global economic burden of NCDs and the role of “best buys”

The substantial global economic costs associated with NCDs were highlighted, particularly in regions like the Gulf Cooperation Council (GCC) countries, where direct healthcare expenses reached nearly $50 billion in 2019, with indirect costs adding another $20 billion (9). Modifiable risk factors, such as tobacco use, unhealthy diets, and physical inactivity, are the primary drivers behind this financial and health burden. Despite the availability of “Best Buys,” the implementation of these strategies has been uneven across countries, primarily due to resource limitations, inadequate capacity, and insufficient multi-sectoral collaboration.

“Countries are facing challenges in implementing the ‘Best Buys’ due to limited resources and capacity. We must adopt a whole-of-system approach to make progress,” -Dr. Richter.

The global commitments to reducing premature mortality from NCDs, as enshrined in the United Nations High-Level Meetings and SDG 3, aim to cut premature mortality by one-third by 2030 (10). However, many countries are not on track to achieve this target. The importance of adaptable interventions that fit different political, economic, and social contexts was emphasized, with speakers calling for stronger cross-sectoral collaboration to overcome the obstacles to implementation.

### 4.2 Expanding the “4×4” NCD framework to include mental health and environmental factors

The expanded “5×5” framework builds on the traditional “4×4” model by incorporating mental health and environmental factors such as air pollution (7). Air pollution is a major contributor to NCDs, particularly in urban areas of the EMR and Africa. Additionally, mental health plays a critical role in comprehensive public health strategies, particularly in the EMR and Africa, where countries face competing priorities due to ongoing infectious disease challenges.

“Mental health is an integral part of NCD management, and air pollution is a growing threat in urban areas and beyond. We cannot address NCDs without considering these factors,”- Dr. Hammerich.

The importance of empowering communities, ensuring universal health coverage, and implementing evidence-based strategies like the “Best Buys” was highlighted by speakers. Key interventions include the MPOWER package for tobacco control (11), the SHAKE package for sodium reduction (12), and new accelerators like the HEARTS technical package for hypertension control (13) and cancer prevention through vaccinations.

The concept of NCD “accelerators” was introduced during the discussion, which are initiatives aimed at achieving SDG target 3.4.1—reducing premature mortality from NCDs by one-third by 2030. The accelerators include interventions like hypertension control, cancer prevention through HBV and HPV vaccinations, and the HEARTS technical package (14).

Participants were curious about whether the “5×5” framework would be expanded in the future, potentially to a “6×6” model. Speakers responded that future expansions may be considered if additional priorities emerge. However, the current framework is robust and adaptable, offering a comprehensive foundation to effectively address a wide range of NCD-related challenges.

“The ‘5×5’ framework is adaptable and provides a solid foundation for addressing various health challenges,” Dr. Richter remarked, signaling that further expansions would be considered as necessary but are not immediately planned.

### 4.3 Strengthening multi-sectoral collaboration for NCD prevention and control

A recurring theme across the presentations was the need for stronger multi-sectoral collaboration to effectively address NCDs. Speakers highlighted the importance of actively involving sectors beyond health, such as education, urban planning, and finance, in efforts to combat NCDs. Addressing the social and environmental determinants of health requires a broad, coordinated approach across governments and communities.

Countries were urged by the speakers to strengthen their leadership, improve multi-sectoral collaboration, and advocate for the global prioritization of NCDs in upcoming international meetings, including the United Nations General Assembly. A concerted effort involving governments, international organizations, and communities is necessary to overcome the challenges posed by NCDs and to seize the opportunities for improving public health outcomes in these regions.

“Changing behaviors around salt and sugar consumption is often difficult, and requires strong political leadership and collaboration across sectors,” Dr. Hammerich noted, indicating that effective regulation is crucial for success.

Speakers called for a whole-of-system approach, involving various sectors to effectively implement the “Best Buys” and achieve the SDGs by 2023. While the “Best Buys” offer countries a pathway to prioritize investments in NCD prevention, their successful implementation requires dedicated technical expertise, resources, and robust engagement from stakeholders across multiple sectors.

This sentiment was echoed by Africa CDC, which is working to align Member States and regional organizations to coordinate action across sectors. The importance of multi-sectoral action to address both the commercial determinants of health and the rising burden of mental health issues is stressed in African CDC’s comprehensive strategy that focuses on NCDs, injuries, and mental health promotion. This strategy leverages existing policies and frameworks, promoting a multi-sectoral approach tailored to the unique needs of African nations. It aims to strengthen health systems, build capacity, and foster collaboration at the continental level (15).

“NCDs and mental health require collaboration across all sectors—no one sector can do this alone,” Dr. Kakunze.

These observations align with existing literature that emphasizes the importance of multi-sectoral collaboration, such as the WHO’s “Health in All Policies” (HiAP) approach, which has been shown to improve policy coherence and facilitate the implementation of NCD interventions across sectors (16, 17).

### 4.4 The need for sustainable financing and access to medicines

Sustainable financing emerged as a critical theme. The challenge of securing long-term funding for NCD prevention and mental health programs was emphasized by Dr. Kakunze, who called for the establishment of a regional African Fund, modeled after the Global Fund, to support these efforts. Africa CDC’s efforts to improve access to affordable NCD and mental health medicines and diagnostics through local manufacturing and pooled procurement initiatives were discussed.

“Without sustainable financing, we cannot build the systems needed to address NCDs and mental health in the long term,” Dr. Kakunze remarked, highlighting the need for innovative funding solutions at both regional and continental levels.

Africa CDC is working to strengthen the capacity of Member States to provide equitable access to essential medicines and technologies, ensuring that cost-effective interventions reach the populations that need them the most.

### 4.5 Building workforce capacity for NCD and mental health management

Another prominent theme was the need to build and strengthen the health workforce to manage NCDs and mental health issues effectively. Africa CDC supports task-shifting models, where healthcare workers are trained to deliver NCD and mental health services, particularly in resource-constrained settings. The importance of building communities of practice and fostering peer learning to strengthen healthcare systems across Africa was highlighted.

“We must build the capacity of our health workforce to manage NCDs and mental health effectively, especially in resource-limited settings,” Dr. Kakunze.

A key initiative mentioned was the Africa CDC Mental Health Leadership Programme (18) and the creation of FETP focused on NCDs and injuries, designed to equip health workers with the skills needed to manage these complex health issues.

Similar workforce challenges and task-shifting models have been reported in previous studies across sub-Saharan Africa, supporting the feasibility and impact of such approaches in resource-limited contexts (19, 20).

### 4.6 Strengthening health systems and advocacy for NCDs and mental health

All speakers underscored that the success of “Best Buy” interventions depends heavily on their adaptability to the diverse political, economic, and social contexts within different countries. These interventions, though cost-effective and evidence-based, cannot be implemented uniformly across all regions. Tailoring the approach to suit each country’s unique landscape is essential for successful adoption and sustainability.

Africa CDC is enhancing health systems across the continent, with a focus on strengthening the capacity of Ministries of Health and National Public Health Institutes. It is actively developing national frameworks to tackle NCDs, injuries, and mental health challenges. Key areas of focus include improving surveillance systems, bolstering risk communication, and fostering community engagement, ensuring that these frameworks are integrated into the broader health systems.

The continued need for strong advocacy efforts at both national and continental levels was emphasized. Political commitment to NCDs and mental health is crucial for sustained progress. The COVID-19 pandemic highlighted the importance of a coordinated response to health crises, reinforcing the need for robust structures and cooperation to address the rising burden of NCDs, injuries, and mental health issues. The pandemic serves as a call to action for more unified and resilient health systems in the region.

### 4.7 Balancing funding between communicable and non-communicable diseases

The challenge of underfunding for NCDs was highlighted by speakers, especially in low- and middle-income countries (LMIC), where resources are often focused on communicable diseases. Speakers argued that prevention strategies are usually more cost-effective than disease management, and integrating NCD prevention into existing healthcare systems can help balance this disparity.

This observation reinforces global findings that prevention-focused investment yields higher cost-effectiveness and long-term impact than reactive disease management, especially in LMICs (17, 21).

“Prevention is more cost-effective than disease management. Simple interventions, like routine blood pressure measurements, can integrate NCD prevention into existing services,” Dr. Richter.

The importance of engaging communities and promoting healthy lifestyles to reduce the NCD burden effectively was stressed.

### 4.8 Recommendations

Table 1 highlights the recommendations from this roundtable.

**Table 1:** Roundtable recommendations.

Recommendation	Description	Supporting literature
Strengthen multi-Sectoral collaboration	Governments must actively involve sectors beyond health—such as education, finance, urban planning, and agriculture—to address the social and environmental determinants of NCDs. A “whole-of-system” approach is essential for implementing the “Best Buys” and achieving the SDGs.	(16, 17, 28, 30)
Tailor interventions to local contexts	NCD interventions should be adaptable to the unique political, economic, and social contexts of each country. This includes aligning efforts with national priorities and tailoring strategies to available resources and healthcare infrastructure.	(4, 6, 23)
Enhance political commitment and advocacy	Strong political will and advocacy at both national and international levels are crucial for prioritizing NCDs within health agendas. Governments must take leadership in advancing NCD prevention and control, ensuring these efforts are integrated into broader public health policies.	(4, 5, 36, 39)
Secure sustainable financing	Establish innovative funding mechanisms, such as a regional African Fund for NCDs and mental health, to ensure long-term support for prevention and treatment programs.	(17, 31, 32)
Focus on workforce development	Building and strengthening the healthcare workforce, particularly through task-shifting and capacity-building initiatives, is necessary to manage NCDs and mental health issues in resource-constrained settings. Expanding programs like the FETP for NCDs and injuries can provide the needed expertise.	(19, 20)
Address the mental health and environmental dimensions of NCDs	Mental health and environmental factors like air pollution are critical components of NCD management. Countries should incorporate these into national NCD strategies, ensuring that mental health is prioritized in public health interventions.	(7)
Leverage the COVID-19 experience	Build on the health system structures and collaborations established during the COVID-19 pandemic to strengthen the response to NCDs. This includes improving surveillance systems, enhancing risk communication, and fostering community engagement for NCD prevention and control.	(30, 37)

## 5 Discussion

The findings from the roundtable highlight critical challenges and opportunities for advancing the NCD agenda. While progress has been made, significant gaps remain in the effective implementation of cost-effective interventions, particularly in resource-limited settings. The discussion emphasized the importance of adaptability, multi-sectoral collaboration, sustainable financing, and workforce development, while also exploring the broader implications for public health policy.

One of the central themes of the roundtable was the importance of adapting WHO’s Best Buy interventions to the specific political, economic, and social contexts of individual countries. The success of these interventions depends on their flexibility, as they cannot be uniformly implemented across diverse regions like the EMR and Africa. The variability in healthcare infrastructure, financial resources, and political will among countries necessitates a tailored approach to intervention delivery (22).

The importance of modifiable risk factors—such as tobacco use, unhealthy diets, and physical inactivity—in driving NCDs was underscored during the workshop. However, the uneven implementation of Best Buys due to limited resources and insufficient multi-sectoral collaboration remains a critical barrier. Tailored interventions, when aligned with local political and economic realities, achieve better health outcomes (23).

Effective NCD prevention and control require collaboration across multiple sectors (24), as emphasized by speakers. Addressing the social determinants of health, such as education, urban planning, and food production, requires concerted efforts from sectors beyond healthcare (25). Conflicts between economic interests (e.g., trade and agriculture) and health priorities (e.g., reducing sugar and salt consumption) pose significant challenges to implementing health policies. Strong national leadership and effective negotiation skills are necessary to align these competing interests (26, 27).

The roundtable echoed findings from other global public health efforts, which show that multi-sectoral approaches lead to more sustainable interventions (28). The integration of NCD prevention with national development agendas in LMICs requires sectors such as finance and urban planning to be engaged to address the underlying causes of NCDs effectively (23, 29). In particular, WHO’s “Health HiAP approach has been shown to improve policy coherence and ensure that health is considered in decisions across different sectors in several countries (16, 30).

The issue of financing emerged as a critical barrier to NCD prevention and control in both the EMR and Africa. Dr. Adelard Kakunze’s highlighted the urgent need for sustainable financing mechanisms, calling for the creation of a regional “African Fund,” similar to the Global Fund against AIDS, tuberculosis, and malaria, dedicated to NCDs and mental health issues. Securing long-term funding for NCD programs is essential for building resilient health systems capable of addressing the growing burden of NCDs in these regions (31).

The economic costs associated with NCDs and mental illness are significant. For instance, in 2011, the total economic loss from NCDs in LMICs was projected to exceed $21.3 trillion by 2030 if no substantial interventions are made (32). Investing in prevention, including routine screening and public health campaigns, offers a high return on investment (17, 33). The point highlighted during the discussion about the cost-effectiveness of prevention strategies, such as routine blood pressure monitoring, aligns with global estimates indicating that every dollar invested in NCD prevention yields significant health and economic benefits (34).

The roundtable discussion also emphasized the need to build capacity within the healthcare workforce to manage NCDs and mental health issues. Dr. Kakunze noted Africa CDC’s focus on task-shifting models, where community health workers and lower-level healthcare staff are trained to provide NCD and mental health services. Task-shifting is especially relevant in resource-constrained settings where healthcare workforce shortages are prevalent (19). Studies from sub-Saharan Africa have shown that task shifting can improve health outcomes while reducing the burden on overextended healthcare systems (20). Furthermore, the FETP for NCDs and injuries, mentioned by Dr. Kakunze, is an important initiative that will help build the technical expertise required to manage the growing NCD burden across the continent.

The roundtable provided a platform for discussing actionable recommendations for policymakers. A whole-of-system approach, including multi-sectoral collaboration, political commitment, and community engagement, is essential for the successful implementation of NCD strategies (35). National governments must prioritize NCDs within their broader health agendas, ensuring that interventions are integrated into existing healthcare systems. Additionally, advocating for NCDs at high-level global forums—such as the United Nations General Assembly—will be crucial in garnering political and financial support (36).

The COVID-19 pandemic has underscored the need for resilient health systems capable of addressing both communicable and non-communicable diseases (37). Building on the structures established during the pandemic, as Dr. Kakunze pointed out, will allow countries to strengthen their responses to NCDs and mental health issues. Additionally, expanding global partnerships and securing sustainable funding mechanisms will be pivotal in addressing the rising burden of NCDs, especially in LMICs (38).

This manuscript presents insights from a high-level regional roundtable and is supported by relevant literature; however, several limitations must be acknowledged. The findings reflect the perspectives of a selected group of invited public health professionals and may not fully capture the diversity of views across all countries in the Eastern Mediterranean and African regions. As the synthesis is derived from a single event, it may not encompass the full range of NCD implementation experiences or national policy dynamics. Additionally, the discussion is informed by summarized presentations and expert dialogue rather than in-depth empirical data, which introduces potential selection and information bias.

Furthermore, variability in the quality, completeness, and frequency of NCD reporting, particularly in LMICs, can influence the accuracy and comparability of data cited in the manuscript. Some of the economic and epidemiological indicators referenced are based on modeled projections, which may not fully reflect real-time developments or context-specific shifts in disease burden. These limitations affect the generalizability of the recommendations. Nonetheless, efforts were made to triangulate the roundtable findings with evidence from global and regional sources, including WHO frameworks and peer-reviewed studies, to ensure their alignment with current public health priorities and best practices.

## 6 Conclusion

The roundtable reinforced that addressing the growing burden of NCDs in the EMR and Africa requires context-specific, adaptable interventions and sustained multi-sectoral collaboration. Although the Best Buy interventions provide an evidence-based, cost-effective framework, uneven implementation persists due to limitations in resources, competing health priorities, and insufficient coordination across sectors. The incorporation of mental health and environmental factors, as seen in the expansion to the “5×5” framework, highlights the evolving nature of NCD challenges and the need for flexible strategies.

To effectively reduce premature mortality from NCDs, political commitment, innovative funding mechanisms, and stronger health systems are essential. The integration of NCD prevention into broader health systems, advocacy for prioritization at national and global levels, and workforce capacity building remain critical to making meaningful progress. The COVID-19 pandemic has underscored the urgency of building resilient health systems capable of addressing both communicable and non-communicable diseases, and leveraging lessons learned from the pandemic response will be pivotal in enhancing NCD prevention and control.

## Data Availability

The original contributions presented in the study are included in the article/supplementary material, further inquiries can be directed to the corresponding authors.
